# Cytokine Levels in Neural Pain in Leprosy

**DOI:** 10.3389/fimmu.2020.00023

**Published:** 2020-01-24

**Authors:** Débora Bartzen Moraes Angst, Roberta Olmo Pinheiro, Joyce Soares da Silva Vieira, Roberta Arnoldi Cobas, Mariana de Andréa Vilas-Boas Hacker, Izabela Jardim Rodrigues Pitta, Louise Mara Giesel, Euzenir Nunes Sarno, Márcia Rodrigues Jardim

**Affiliations:** ^1^Leprosy Laboratory, Oswaldo Cruz Foundation (Fiocruz), Rio de Janeiro, Brazil; ^2^Postgraduate Program in Neurology of Federal University of Rio de Janeiro State (UNIRIO), Rio de Janeiro, Brazil; ^3^Endocrinology Discipline of the Faculty of Medical Sciences, State University of Rio de Janeiro (UERJ), Rio de Janeiro, Brazil; ^4^Neurology Discipline of the Faculty of Medical Sciences, State University of Rio de Janeiro (UERJ), Rio de Janeiro, Brazil

**Keywords:** leprosy, nociceptive pain, neuropathic pain, cytokines, diabetes, neuropathy

## Abstract

Pain is a frequent symptom in leprosy patients. It may be predominantly nociceptive, as in neuritis, or neuropathic, due to injury or nerve dysfunction. The differential diagnosis of these two forms of pain is a challenge in clinical practice, especially because it is quite common for a patient to suffer from both types of pain. A better understanding of cytokine profile may serve as a tool in assessing patients and also help to comprehend pathophysiology of leprosy pain. Patients with leprosy and neural pain (*n* = 22), neuropathic pain (*n* = 18), neuritis (nociceptive pain) (*n* = 4), or no pain (*n* = 17), further to those with diabetic neuropathy and neuropathic pain (*n* = 17) were recruited at Souza Araujo Out-Patient Unit (Fiocruz, Rio de Janeiro, RJ, Brazil). Serum levels of IL1β, IL-6, IL-10, IL-17, TNF, CCL-2/MCP-1, IFN-γ, CXCL-10/IP-10, and TGF-β were evaluated in the different Groups. Impairment in thermal or pain sensitivity was the most frequent clinical finding (95.5%) in leprosy neuropathy patients with and without pain, but less frequent in Diabetic Group (88.2%). Previous reactional episodes have occurred in patients in the leprosy and Pain Group (*p* = 0.027) more often. Analysis of cytokine levels have demonstrated that the concentrations of IL-1β, TNF, TGF-β, and IL-17 in serum samples of patients having leprosy neuropathy in combination with neuropathic or nociceptive pain were higher when compared to the samples of leprosy neuropathy patients without pain. In addition, these cytokine levels were significantly augmented in leprosy patients with neuropathic pain in relation to those with neuropathic pain due to diabetes. IL-1β levels are an independent variable associated with both types of pain in patients with leprosy neuropathy. IL-6 concentration was increased in both groups with pain. Moreover, CCL-2/MCP-1 and CXCL-10/IP-10 levels were higher in patients with diabetic neuropathy over those with leprosy neuropathy. In brief, IL-1β is an independent variable related to neuropathic and nociceptive pain in patients with leprosy, and could be an important biomarker for patient follow-up. IL-6 was higher in both groups with pain (leprosy and diabetic patients), and could be a therapeutic target in pain control.

## Introduction

Despite ongoing efforts to eradicate leprosy in Brazil, it remains an endemic disease and a public health challenge (1). Nerve trunk involvement in leprosy results in debilitating deformities in 20% of all patients (2). The accompanying neural pain, experienced by up to 70% of these patients, presents as nociceptive (neuritis) or neuropathic pain resulting from damage or disease of sensory pathway (2–5). Currently, pain is a functional disability not regarded an inability when leprosy patients are monitored by the Brazilian Department of Health. As a result, the disability of leprosy patients may be underestimated.

Leprosy is a chronic infectious disease caused by *Mycobacterium leprae*, an intracellular pathogen that preferentially infects macrophages and Schwann cells.

*Mycobacterium leprae* alters mitochondrial glucose metabolism in Schwann cell (SC). This affects the complicated modulation of Schwann cell and axons, resulting in a reduction of axonal metabolism, demyelination, and loss of axons (6).

Schwann cells also play an important role in pain modulation. SC can proliferate and secrete soluble mediators which control Wallerian degeneration and regeneration. Amongst the soluble mediators are pro-inflammatory cytokines that function as chemoattractant, but may also sensitize nociceptors (7).

Some studies have indicated cytokines as possible pain biomarkers. A number of preclinical and clinical studies are being developed (8) by using biomarkers in a correlation with patients with pain. For example, IL-6 is a prominently pro-inflammatory cytokine secreted by mast cells, macrophages, lymphocytes, neurons, and glial cells (9). Under certain conditions, however, it can modulate anti-inflammatory responses (10).

In animal models, IL-6 has been shown to mediate neuropathic pain development (11). In fact, some studies have demonstrated that patients with neuropathic pain due to intervertebral disc herniation or the carpal tunnel syndrome had increased serum IL-6 and TNF (12, 13). Similar reports of increased serum IL-6 have occurred in patients with post-herpetic neuralgia, which have also correlated quantitatively with pain intensity in neuralgia (14). In rats, TNF seems to be responsible for the neuropathic pain caused by nerve injury (15). In animal models of neuropathic pain, the involvement of proinflammatory cytokines such as TNF, IL-1β, and IL-6 after peripheral nerve involvement has been well-documented (15, 16). Regional complex pain syndromes, peripheral neuropathy, and neuropathic pain associated with spinal cord injury are known to be associated with increased serum IL-6 and TNF levels (17–19).

IL-1β is a pluripotent cytokine produced and secreted under conditions of stress by immune cells including macrophages, monocytes, and microglia (9). This cytokine is one of many agents involved in neuropathic pain, and its production may also be related to the presence of specific immunological markers (4).

A study with rats and mice undergoing transient focal demyelination of sciatic nerve have reported increased expression of CCL-2/MCP-1 and CXCL-10/IP-10 receptors (20).

Although prior studies have investigated pain in leprosy (2, 21, 22), no study has currently provided alternatives to better differentiate nociceptive from neuropathic pain.

In addition, the evaluation of cytokines in most studies was limited to the pain resulting from acute inflammatory episodes known as leprosy reactions. However, high levels of pro-inflammatory cytokines during a reaction episode can mistake the accurate understanding of the mechanisms involved in leprosy pain. Furthermore, the treatment of pain is not specific, highlighting the need of studies focusing on the examination of neural pain mediators and mechanisms. The present report has investigated the cytokine profile in serum samples of leprosy patients with pain.

## Methodology

### Study Design

This retrospective cross-sectional study is based on data collected from Souza Araujo Out-Patient Unit (ASA) (Fiocruz, Rio de Janeiro, RJ, Brazil) and Diabetes Outpatient Clinic of Pedro Ernesto University Hospital (State University of Rio de Janeiro, Rio de Janeiro, RJ, Brazil). Medical records and a database of leprosy neuropathy patients evaluated at ASA from January 1998 to December 2017 were also the source of data collection, together with data regarding histopathology of nerve biopsy to determine neuropathy etiology. During the aforementioned period of time, 662 biopsies were performed. Within the biopsied patients, 311 were diagnosed with leprosy. Out of the 311 leprosy patients, 89 had pain during the evaluation prior to nerve biopsy, while 222 had no pain. All the biopsied patients having confirmed leprosy neuropathy in combination with neural pain were selected to the study. The other patients were selected to take part into two comparative groups, namely, one group consisting of painless leprosy neuropathy patients, the other group consisting of patients with diabetic neuropathy in combination with neuropathic pain. The total number of patients was divided up into three groups, as follows:

Group A: Leprosy Neuropathic Pain Group (89 patients); Group B: Painless Leprosy Neuropathy Comparative Group (50 patients); and Group C: Diabetic Neuropathic Pain Comparative Group with Type II Diabetes Mellitus (23 patients).

Patients with comorbidities known to cause peripheral neuropathy such as rheumatologic diseases, alcoholism, hypothyroidism, diabetes (except Group C patients), B12 hypovitaminosis, HIV or viral hepatitis, patients in corticosteroid treatment or in reaction, further to patients with incomplete medical records or without a laboratory-stored blood sample were excluded from this study. Accordingly, 106 patients were excluded. Out of them, 67 patients were excluded from Group A, 33 patients were excluded from Group B, and 6 patients from Group C. After applying the exclusion criteria, 56 patients have remained in the study: 22 in Group A; 17 in Group B; and 17 in Group C.

**Figure 1:**
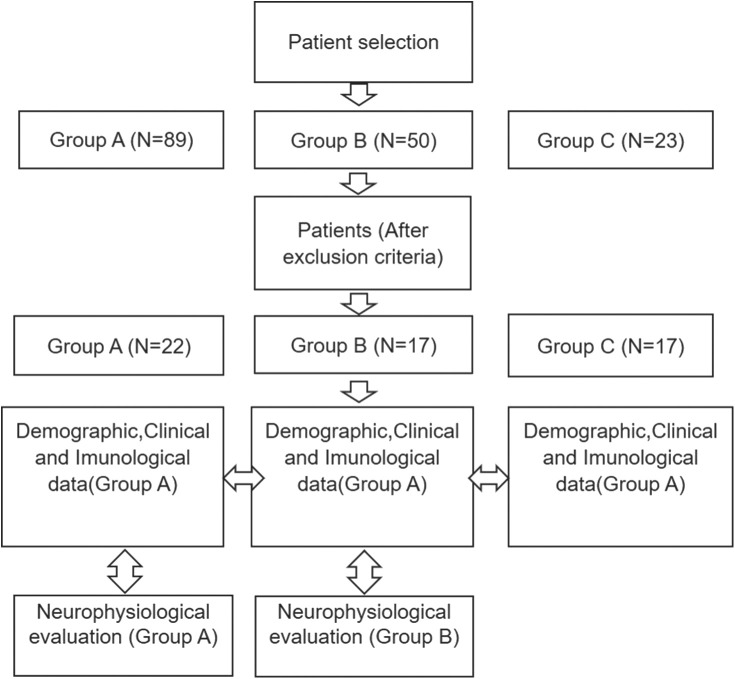
Methodology flowchart. Group A: Leprosy neuropathic pain group; Group B: painless leprosy neuropathy comparative group; Group C: diabetic neuropathic pain comparative group with type II diabetes mellitus.

The following flowchart describes the methodology used in the present study (Figure 1). As soon as the inclusion and exclusion criteria were met, epidemiological, clinical, immunological, and neurophysiological data were collected. Epidemiological, clinical, and immunological data were obtained from all patients. Neurophysiological data were not collected from diabetic patients, considering these patients were not submitted to such examinations.

### Case Definitions

Neuropathic pain was defined as pain caused by damage or disease affecting the somatosensory nervous system, according to International Association for the Study of Pain. Neuropathic pain diagnosis was based on European Federation of Neurological Societies (EFNS) guidelines (23). Patients were selected in case of pain classified as probable or definite. “Probable” neuropathic pain requires supporting evidence obtained from a clinical examination of sensory signs. Probable criteria was confirmed by physical examination. “Definite” neuropathic pain requires an objective diagnostic test to confirm the somatosensory nervous system lesion or disease. Definite criteria was confirmed by electromyography. All patients having neuropathic pain in combination with leprosy have filled-in definite criteria. All patients having neuropathic pain in combination with diabetes have filled-in probable criteria. Nociceptive pain was defined as the pain resulting from nociceptor activation, secondary to tissue damage or potential tissue-damaging stimuli. Nociceptive pain is the most important pathological mechanism related to neuritis, defined as the presence of one or more nerves with enlargement, pain, or loss of function (24).

### Clinical Evaluation

Information on the neurological examination performed prior to nerve biopsies was gathered from medical records. The type of pain (stinging, burning, electric shocklike, cold, other), pain intensity (numerical pain rating scale from 0 to 10 or 11 point scale) (25), and pain location were recorded. Furthermore, the presence of neural thickening, previous reactional episodes (type I or II), further to information on sensitive and motor neurological examination were gathered from database.

Type 1 reaction or reversal reaction (RR) is a type IV delayed hypersensitivity reaction characterized by ulcerative, red, swollen skin lesions followed by fever (26). Type II reaction, or erithema nodosum leprosum (ENL), is an acute inflammatory condition, characterized by nodules and painful, raised red papules. These nodules are accompanied by neuritis, uveitis, iridocyclitis, episcleritis, arthritis, dactilitis, lympohadenitis, and/or orquitis. Fever, prostration, anorexia and other constitutional symptoms are frequent (27).

### Neurophysiological Evaluation

Data were collected from examinations performed via a 4-channel Nihon-Koden-Neuropack S1 equipment, in accordance with standard procedures (28). Amplitude, velocity, and latency were recorded for the median, radial, ulnar, and sural sensory nerves, further to the median, ulnar, and peroneal motor nerves.

According to the results, the following six pathophysiological classifications were determined:

- No injury, or normal: when the findings were within the reference values;
- Axonal injury: when there was a sharp decrease in compound muscle action potential (CMAP) amplitude (more than 30% of the lower limit), or a moderate decrease with a slight reduction in conduction velocity (>70% the lower limit of normality), or a slight prolongation of latency (<130% the upper limit of normality);
- Demyelinating lesion: when there was a sharp decrease in conduction velocity (below 70% the lower limit of normal), or an evident prolongation of CMAP latencies (>130% the upper limit of normal) with slightly reduced amplitude including the presence/absence of demyelination markers such as conduction block (CB) and temporal dispersion (TD);
- Demyelinating lesion with secondary axonal damage, or mixed lesion: when there was either axonal or demyelinating impairment, i.e., sharp decrease in amplitudes with greatly reduced velocities and quite prolonged latencies, further to demyelination markers, such as CB and TD, with a sharp reduction in amplitudes;
- Not fulfilled: sensory or motor alterations that did not fit the criteria above; and
- Unclassified/unresponsive: in case of absent sensory and motor responses (29).

The clinical and neurophysiological diagnosis of neuropathy was defined as a clinically or neurophysiologically detectable impairment of sensory and/or motor nerve.

### Histopathological Evaluation

The selection of which sensory nerves to be biopsied in each patient was based on the findings regarding their clinical and neurophysiological involvement. Nerve biopsies were performed in accordance with institutional protocol and leprosy etiology in pure neural leprosy patients, and confirmed by Antunes et al. (30).

### Serum Cytokine Levels

Serum samples used to measure cytokine concentrations were stored at −70°C, according to Good Laboratory Practices. Serum concentrations of IL1-β, IL-6, IL-10, IL-17, TNF, CCL-2/MCP-1, IFN-γ, CXCL-10/IP-10, and TGF-β in the samples were evaluated via ELISA, as specified by the manufacturer (eBioscience-San Diego, CA, United States). Serum levels were measured in picograms per mL (pg/mL).

### Data Analysis

All the collected patient data were recorded on the database spreadsheets commonly used in outpatient clinic. Data analysis was performed using SPSS statistics 22 program. A descriptive analysis of explanatory variables described below was performed. Comparisons between groups were carried out by means of Chi-square and Fisher's tests for categorical variables, and Kruskal–Wallis test for continuous variables. A logistic regression using a stepwise method was performed to evaluate any possible pain variables.

Explanatory Variables:

The following variables were analyzed:

- Demographic data: age (in years), gender (female or male), and ethnicity (white, brown, or black);
- Clinical data: clinical form of leprosy (in groups A and B) according to Ridley and Jopling (31) criteria, pain intensity (by means of the numerical pain scale from 0 to 10, wherein 0 is the absence of pain and 10 refers to the most intense one), pain characteristics (burning, stinging, electric shocklike, stabbing), pain extension (localized in up to two or more nerves), the presence of neural thickening, reactional episodes, sensory alterations according to the size of affected fibers (small or large involvement), and motor alterations;
- Nerve conduction study: lesion pattern (axonal, demyelinating, demyelinating with secondary axonal degeneration, not fulfilled, or unclassified/unresponsive);
- Cytokine and chemokine serum concentrations: The concentrations of IL1-β, IL-6, IL-10, IL-17, TNF, CCL-2/MCP-1, IFN-γ, CXCL-10/IP-10, and TGF-β were recorded in picograms per mL (pg/mL).

### Ethical Considerations

Our research was carried out in compliance with the International Compilation of Human Research Standards, and approved by the Ethics Committee of Oswaldo Cruz Foundation. Approval number: 2.972.967 CAAE 94630718.7.0000.5248. All patients have signed informed consent before any procedure.

## Results

### Demographic Characteristics

Demographic data of the three Groups are described in Table 1. The mean age was significantly higher in type II Diabetes Group than in Leprosy Group. There was no statistically significant difference between the mean ages in Leprosy Groups with or without pain (45 and 47 years old, respectively).

**Table 1:** Demographic data of group A (leprosy neuropathy with neural pain), group B (leprosy neuropathy without pain), group C (diabetic neuropathy with neuropathic pain).

**Demographic data**		**Group A**	**Group B**	**Group C**	***p*-Value**
Gender	Female	9 (40.9%)	8 (47.1%)	9 (52.9%)	0.755
	Male	13 (59.1%)	9 (52.9%)	8 (47.1%)	
Ethnicity	White	9 (42.9%)	10 (58.8%)	6 (35.3%)	0.482
	Brown	5 (23.8%)	1 (5.9%)	3 (17.6%)	
	Black	7 (33.3%)	6 (35.3%)	8 (47.1%)	
Age	Mean (years)	45.2	47.4	74.0	<0. 00001

### Clinical Characteristics

A history of previous reactional episodes was noted in 6 patients (27.3%) from Group A and none from Group B, a statistically significant finding (*p* = 0.019). Neural thickening was noted in both Groups at a similar frequency. Out of patients from Group A, 18 (81.8%) had neuropathic pain and 4 had neuritis (Table 2).

**Table 2:** Clinical characteristics of patients with leprosy neuropathy.

**Clinical characteristics**		**Group A**	**Group B**	***p*-Value**
Clinical form	NP	13 (65%)	17 (100%)	N.A
	LL	5 (25%)	0	
	BB	1 (5%)	0	
	BL	1 (5%)	0	
Leprosy reactional episodes	Yes	6 (27.3%)	0	0.027
	No	16 (72.6%)	17 (100%)	
Neural thickening	Yes	8 (36.4%)	6 (35.3%)	0.945
	No	14 (63.5%)	11 (64.7%)	

*N.A, not applicable; NP, neural pure leprosy form; LL, lepromatous leprosy form; BB, boderline borderline form; BL, boderline lepromatous form*.

As to pain characteristics among leprosy patients, the most frequently mentioned was a burning sensation (50%), followed by pain similar to an electric shock (40.9%). Pain was considered severe (intensity higher than 7) in 81% of all cases; and most of patients have experienced pain in more than 2 nerves according to their neuroanatomical distribution. These findings are summarized in Table 3.

**Table 3:** Description of neural pain characteristics in group A (*N* = 22).

Intensity	Mean	7.95 (±2.20)
	Severe (more than 7)	17 (81%)
Type of pain	Burning Sensation	11 (50%)
	Electric Shock Sensation	9 (40.9%)
	Other	2 (9%)
Number of nerves affected by pain	Less than 2 nerves	9 (40.9%)
	More than 2 nerves	13 (59.1%)

Nerve thickening was present in 36.4% of Group A and 35.3% of Group B patients. Sensory changes characterized by small fibers impairment (such as impaired thermal and pain sensitivities) were present in 95.5% (21 patients) of Group A, 88.2% (15 patients) of Group B, and 70.6% (12 patients) of Group C. Large fibers involvement (characterized by impaired vibration sensitivity) was noted in 18.2% (4 patients) of Group A, 11.8% (2 patients) of Group B, and 100% (17 patients) of Group C. The involvement of large-caliber fibers was significantly higher in Diabetic Group (*p* < 0.00001), whereas there was no statistically significant difference between Groups A and B (*p* = 0.883 and *p* = 0.582, respectively). Motor impairment was noted in 59.1% of Group A and 47.1% of Group B (*p* = 0.455) (Table 4).

**Table 4:** Principal neurological examination in patients with leprosy neuropathy with (Group A) and without pain (Group B).

**Nerve involvement**		**Group A**	**Group B**	***p*-Value**
		***N* = 18**	***N* = 17**	
Small fiber	Yes	21 (95.5%)	15 (88.2%)	0.426
	No	1 (4.5%)	2 (11.8%)	
Large fiber	Yes	4 (18.2%)	2 (11.8%)	0.582
	No	18 (81.8%)	15 (88.2%)	
Motor Involvement	Yes	13 (59.1%)	8 (47.1%)	0.455
	No	14 (63.6%)	11 (64.7%)	

### Serum Cytokines in Patients With Pain

Mean values for IL-1β, TNF, TGF-β, and IL-17 cytokine concentrations were higher in the Group of leprosy neuropathy in combination with neural pain than in the other Groups. Regarding mean values, IL-10 concentrations in Group A were lower than in Groups B and C, with a statistically significant difference between Groups A and C (*p* = 0.001). IL-1β concentrations were significantly higher in Group A than in Groups B and C (*p* = 0.0001 in both comparisons). No difference was noted between Groups B and C (*p* = 0.46).

IL-6 concentrations were higher in patients with diabetic neuropathy in combination with neuropathic pain, further to those with leprosy neuropathy in combination with neural pain in a comparison with leprosy neuropathy patients without pain. This difference was considered statistically significant (*p* = 0.041). Even so, no difference was found between Groups A and C (*p* = 0.75); and TNF was higher in Group A than in Group B. There was no statistical difference between Groups A and B (*p* = 1.0), however Groups A and C were significantly different (*p* = 0.02). Mean values of IL-17 were significantly higher in Group A than in Group B (*p* = 0.01). However, there was no statistical difference in the other group comparisons, although CCL-2/MCP-1 mean values had been higher in Group C than in the other Groups. There was a significant difference when comparing CCL2/MCP-1 values in Group C to Groups A and B (*p* = 0.001 and *p* = 0.01 respectively). No difference was noted between Groups A and B; but the differences between IFN-γ values in Group C (*p* = 0.0001) were higher and significant when compared to the ones in Group A.

**Figure 2:**
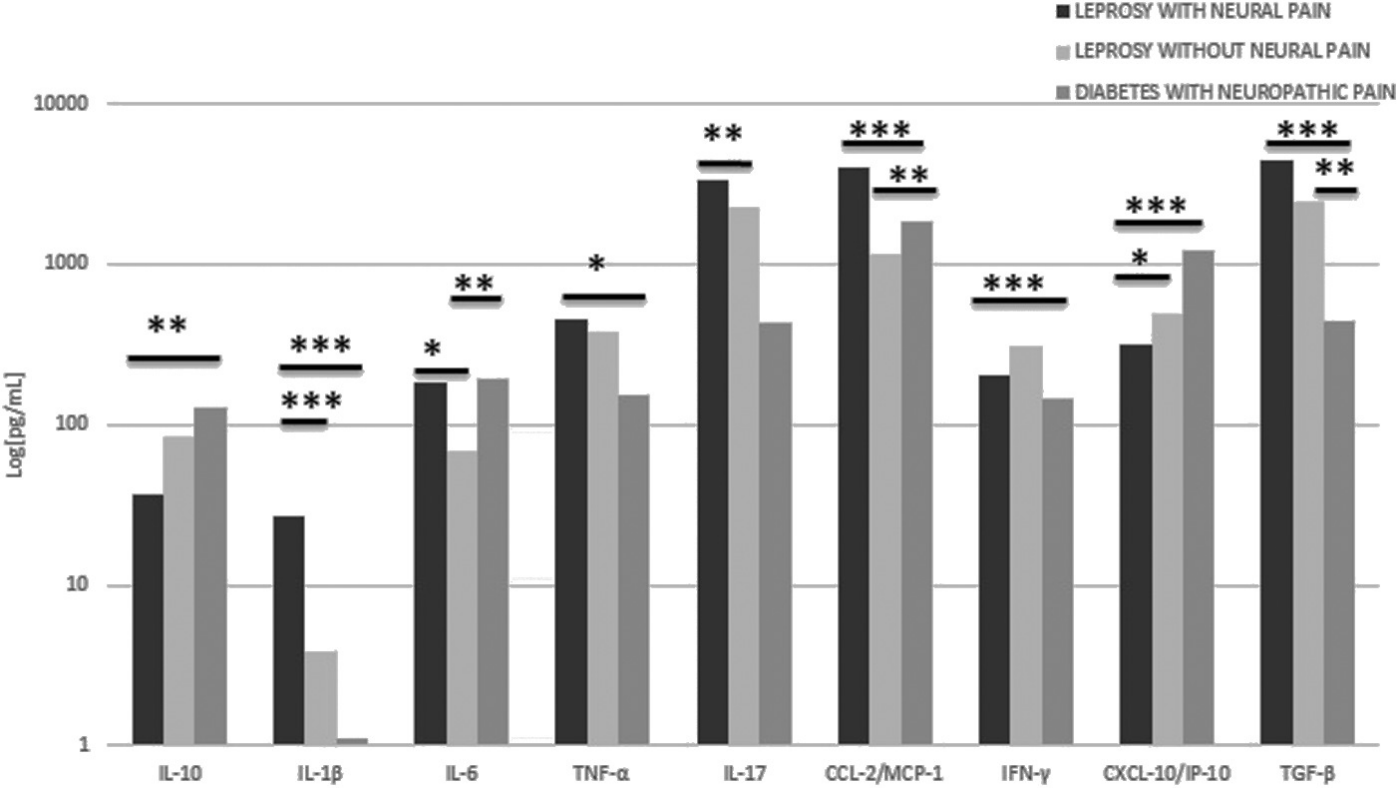
Mean values of serum cytokines in all groups. The graph shows the very high concentration of serum IL-1b levels in the neural pain leprosy group compared to the other groups. Statistically significant differences between groups with leprosy with and without pain were also found regarding IL-6, IL-17, CCL-2/MCP-1, CXCL-10/IP-10 concentrations. *p < 0.05, **p < 0.005, and ***p < 0.0005.

CXCL-10/IP-10 concentrations were higher in Diabetes Group, intermediate in Group B, and lower in Group A. These data were statistically significant between Groups A and B (*p* = 0.02) and Groups A and C (*p* = 0.0001). The opposite have occurred with regard to TGF-β concentration levels, which were higher in Group A and significant between both Groups A and B (*p* = 0.01), further to Groups A and C (*p* = 0.0001). Figure 2 shows the differences in cytokine serum concentrations between the Groups and their significance in each comparison. Logistic regression was performed to Groups A and B regarding variables, age, clinical form, presence of previous history of reaction, nerve conduction pattern, and serum concentration levels of cytokines. IL-1β levels plays the rule of an independent variable when comparing Groups A and B.

In Neural Pain Group, 18 patients had neuropathic pain and 4 had neuritis. Serum concentrations were averaged in patients with neuropathic pain and neuritis. Similar mean concentrations were found for IL-17, IL-10, IL-6, IL-1β, TNF, and TGF-β cytokines. The concentrations of CCL-2/MCP-1, IFN-γ and, CXCL10 / IP-10 were slightly higher in patients with neuropathic pain, as opposed to those with neuritis. In view of the small number of participants having neuritis, a mere descriptive analysis of concentrations was performed.

## Discussion

Neural pain is a quite common symptom in leprosy patients. In a cross-sectional study by Santos et al. (32), the 260 participants included in their report were diagnosed with leprosy; out of them, 195 patients (75%) presented with pain during evaluation, resulting in a lower life quality index. Considering these data as a whole, leprosy pain is definitely a public health problem. Data on the prevalence of neuropathic pain in leprosy fluctuates widely, from 11 to 78.9%, depending on the study (3, 4, 33). The presence of neuropathic pain depends on the moment it is detected, the antimicrobial treatment situation, and the clinical form of the disease (2, 5). In the present study, neural pain prior to the diagnostic biopsy was evidenced in 89 out of the 311 (28.6%) patients treated for leprosy.

Chances are that neuropathic pain is even more common subsequently to multidrug treatment (MDT), as demonstrated by a different kind of study (4, 5, 34). A study by Nascimento et al. (35) has reported cases in which symptoms of neuropathic pain have begun many years after the end of treatment. Another study has traced the slow development of symptoms in a group of six patients who had already finished treatment at least 10 years before the appearance of any typical sensory signs or symptoms (36).

Previous reactional episodes were not noted in Painless Neuropathy Group, but in the Group of leprosy neuropathy in combination with neural pain, which consists mostly of multibacillary patients. Many authors believe that the reactional episodes accompanying leprosy are among the risk factors for neuropathic pain (2, 4, 22, 32, 37).

In the present study, during neurological examination, small fiber neuropathy was the most frequent finding among leprosy patients. These data are similar to the ones previously described (2, 32). Unmyelinated and poorly myelinated fibers could be affected (38). Small fiber neuropathy correlates with neuropathic pain by means of physiology (39, 40). On the other hand, large-caliber sensory fiber involvement is uncommon in leprosy neuropathy (2).

As to nerve conduction studies, despite the absence of statistically significant differences between the groups with and without pain, there was a tendency toward prevalence of demyelinating form in the group with pain. Jardim et al. (41) have described that, in the early stages of infection, nerve conduction alterations of demyelination are commonly noted. The remarkable absence of evocative sensory and motor responses in the group of patients without pain denotes more severe nerve impairment in this group.

Many studies have shown that cytokines are higher in leprosy type I and II reactional episodes, defined as systemic inflammatory complications in leprosy. One study suggests that TNF and IL-10 could possibly predict the occurrence of type I and II reactions, respectively, while increased IL-1β and IFN-γ might also predict the occurrence of both reactional types acting as biomarkers (42). The increase of cytokine levels during reactional episodes has also been described by other authors (43–45).

Again, the present study ascertains that IL-1β is an independent variable related to neural pain group. Although most patients in the present study have neither presented with acute neuritis nor experienced acute reactional episodes, a process of silent neuritis cannot be ruled out (4/56 patients presented with neuritis). Proinflammatory cytokines such as TNF and IL-1β are effective to directly stimulate and sensitize Aδ fibers and type C fibers (46). In rats with neuropathic pain due to chemotherapy-induced neuropathy, these abnormal, spontaneous discharges of A and C fibers are associated with neuropathic pain pathogenesis (47). Increased levels of IL-1β in leprosy and neural pain patients may also be associated with an analogous mechanism, wherein the increased inflammatory response maintains the nociceptive stimulus by peripheral sensitization.

Moreover, increased levels of TNF, TGF-β, and IL-17 in leprosy and neural pain patients have also been detected. One report has shown that, in leprosy, TNF and TGF-β induce apoptosis in Schwann cells, with a consequent damage to peripheral nerve (48). Although no statistical difference in TNF levels in Groups A and B has been found, a difference was noted between Groups A and C, indicating that such a cytokine may be associated with the pathophysiological mechanisms of nerve damage in leprosy, but not in a non-infectious clinical condition like diabetes. TGF-β was significantly higher in leprosy and neural pain group. Studies have reported an increase of this protein in multibacillary patients (25% of group A). Therefore, such a difference may be related to the clinical form and the extent of nerve damage. Higher TGF-β expression in patients with the lepromatous form of the disease is associated with a higher frequency of apoptosis in the lesions (48, 49), further to fibrogenesis (50).

IL-17 levels were increased in Pain Group. The association between higher IL-17 and pain has been previously described in relation to both nociceptive (51) and neuropathic pains (52–54). This cytokine seems to be involved in the maintenance of neuropathic pain due to the activation of astrocytes and the secretion of proinflammatory cytokines (55).

Lower levels of IL-10, an anti-inflammatory cytokine, were noted in the group of patients with leprosy neuropathy in combination with neural pain. IL-10 causes the negative regulation of proinflammatory cytokines (56), presenting reduced levels in chronic pain (19). However, higher values of IL-10 were noted to patients with neuropathic pain as a result of diabetes. The intake of metformin or the use of insulin may increase IL-10 levels in diabetic patients, so that it may have been a confounding factor. Furthermore, increased IL-6 concentrations in the groups of patients with pain secondary to leprosy and diabetes were noted.

IL-6 has been related to postherpetic neuralgia, neuropathic pain secondary to disc herniation, and the carpal tunnel syndrome (12, 13). It may also be a biomarker for chronic pain (8). IFN-γ levels were surprisingly low in leprosy patients. Increased levels of this cytokine have been previously described in the paucibacillary and pure neural forms (42, 57). High IFN-γ levels were noted within diabetic patients, seeming to be related to the pathophysiology of type II diabetes (58). Higher concentrations were found in patients with neuropathic pain secondary to leprosy as opposed to neuritic patients, when comparing patients with neuropathic pain and neuritis. In a study with rats, increased IFN-γ concentrations have been described as key elements in the pathophysiology of neuropathic pain due to hyperexcitability of dorsal horn (59).

Increased CCL2/MCP-1 and CXCL-10/IP-10 levels were noted in diabetic patients with neuropathic pain. Studies indicate that CCL-2/MCP-1 may regulate the excitability in neurons of dorsal ganglion root (DRG) as it is associated with the development of chronic, painful hypersensitivity states (60, 61). A study in rats and mice undergoing transient focal demyelination of sciatic nerve has demonstrated an increased expression of CCL-2/MCP-1 and CXCL-10/IP-10 receptors (20). Patients with neuropathic pain, secondary to leprosy neuropathy, have also had higher concentrations over patients with neuritis, what may be linked to an analogous mechanism of GER hyperexcitability (62).

Furthermore, the present study reaffirms that the immunological profile of patients with neural pain has shown an increased level of pro-inflammatory cytokines. IL-1β is an independent variable related to neural pain and may be an important biomarker for patient follow-up. The lack of differences in neurological examination may indicate that the cytokine profile is more closely related to pain than to nerve damage itself.

Regarding serum levels comparison within the different groups, increased levels of IL-6 were noted in patients with neural pain secondary to leprosy and type II diabetes mellitus in combination with neuropathic pain, indicating that IL-6 may be a pain biomarker. Anti-IL-6 drugs have been investigated as possible targets to pain control. A new drug called tocilizumab has been used in cases of inflammatory arthritis and acute optic neuritis followed by inflammatory and neuropathic pains, respectively (63, 64). An additional study has demonstrated that the new drug was effective to alleviate depression, fatigue, and pain (65).

Finally, the fact that this is a retrospective study is an important limitation. Many patients were excluded due to lack of data or absence of blood samples, which greatly reduced the sample of patients. Moreover, the fact that data were collected from assessments of different examiners is another limitation.

In conclusion, further prospective studies with larger numbers of patients are needed to confirm the role of cytokines in neural pain. However, this study highlights a possible biomarker for follow-up and brings new perspectives in the management of patients with neural pain in leprosy.

## Data Availability Statement

The datasets generated for this study are available on request to the corresponding author.

## Ethics Statement

The studies involving human participants were reviewed and approved by the Oswaldo Cruz Foundation. The patients/participants provided their written informed consent to participate in this study.

## Author Contributions

DA, ES, MJ, and RP formulated the study questions. DA, MJ, RP, MH, and JV designed the study protocol. DA, RP, JV, RC, MH, IP, LG, ES, and MJ conducted the experiment. ES, MJ, and RP supervised the study. DA and MH analyzed the data. DA drafted the manuscript. ES, MJ, and RP revised the manuscript. All authors contributed to the interpretation of the data, read and approved the final version for publication and agreed to be accountable for all aspects of the work in ensuring that questions related to the accuracy or integrity of any part of the work are appropriately investigated and resolved.

### Conflict of Interest

The authors declare that the research was conducted in the absence of any commercial or financial relationships that could be construed as a potential conflict of interest.
